# SLC1A4: A Powerful Prognostic Marker and Promising Therapeutic Target for HCC

**DOI:** 10.3389/fonc.2021.650355

**Published:** 2021-03-11

**Authors:** Xiaozhen Peng, Ruochan Chen, Shenglan Cai, Shanshan Lu, Yiya Zhang

**Affiliations:** ^1^Department of Dermatology, Xiangya Hospital, Central South University, Changsha, China; ^2^Huaihua Key Laboratory of Research and Application of Novel Molecular Diagnostic Techniques, School of Public Health & Laboratory Medicine, Hunan University of Medicine, Huaihua, China; ^3^Hunan Key Laboratory of Viral Hepatitis, Xiangya Hospital, Central South University, Changsha, China; ^4^National Clinical Research Center for Geriatric Disorders, Xiangya Hospital, Central South University, Changsha, China; ^5^The Higher Educational Key Laboratory for Cancer Proteomics and Translational Medicine of Hunan Province, Xiangya Hospital, Central South University, Changsha, China; ^6^Research Center of Carcinogenesis and Targeted Therapy, Xiangya Hospital, Central South University, Changsha, China

**Keywords:** SLC1A4, immune infiltration, prognostic biomarker, therapeutic target, HCC

## Abstract

SLC1A4, a Na-dependent neutral amino acid transporter, was considered to participate in the various pathobiological process, including tumorigenesis. However, the correlation between SLC1A4 and Hepatocellular Carcinoma (HCC) remains unclear. In our study, integrative bioinformatics and functional profiling were performed to reveal the prognosis and potential function of SLC1A4 in HCC. The results showed that the mRNA and protein levels of SLC1A4 were elevated in HCC, and it was a powerful independent prognostic marker for overall survival (OS). The co-expressed genes analysis and GSEA analysis showed that SLC1A4 was related to cell cycle, metabolism, cancer-related pathway. Furthermore, the functional analysis revealed that silenced SLC1A4 inhibited cell proliferation, migration, cell cycle, and promoted cell apoptosis in HCC. Next, immune analysis showed that SLC1A4 expression was positively associated with immune infiltration and immune-related chemokine expression in HCC. Silenced SLC1A4 evidently reduced these chemokines expression in HCC cells. Finally, drug sensitivity analysis revealed potential five sensitivity drugs for HCC patients with high-expressed SLC1A4. In conclusion, our results suggested that SLCIA4 could be a novel predictor prognosis and immunotherapeutic targets of HCC, and the sensitivity drugs may be effective therapeutic strategy for HCC patients with high-expressed SLC1A4.

## Introduction

HCC, a common type of primary liver cancer (PLC), which accounts for approximately 70–90% of PLC patients globally (1). Among 700,000 new HCC cases annually globally, seventy percent were in China and the disease incidence still increases recently. The major risk factors of HCC include viral hepatitis, alcohol consumption, liver cirrhosis, diabetes, some metabolic diseases and so on (2). For patients in the early stage of HCC, curative means of treatment, including liver transplantation, ablative therapies and surgical resection were considered to improve survival in patients with HCC. In fact, most patients tend to be diagnosed at the late-stage at first presentation. Obviously, the prognosis is poor and the survival rate is low for HCC patients with advanced stage. With the development of immunology and depth study of the mechanism of cancer initiation, HCC treatment goes into the times of molecular targeted therapy and immunotherapy (3). Thus, it is of vital significance to explore novel molecular targets associated with the immune response of HCC.

SLC1A4 (also referred to as ASCT1) belongs to the SLC1 family, which encompasses five high-affinity glutamine transporters and two neutral amino acid transporters. It is a Na-dependent neutral amino acid transporter for threonine, cysteine, serine and alanine (4). SLC1A4 has been identified in neuronal and non-neuronal cells and tissues, including astrocytes, neurons, the digestive system, hepatocytes, and the kidney and adrenal glands. Studies revealed the important role of SLC1A4 in early neuronal development and diseases, such as schizophrenia and visual dysfunction, developmental delay, microcephaly and hypomyelination (5). Glutamine is critical for the immune system and it is required to support optimal lymphocyte proliferation and production of cytokines by lymphocytes and macrophages (6). The knockdown of glutamine transporter decreases in intracellular glutamine concentration and then increases in the proliferation and effector function of cytotoxic CD8+ T cells and led to reduced T-reg proliferation and function (7). The CD8+ T cells plays an important role of antitumor immunity in HCC (8, 9). So, we speculated the important role of SLC1A4 in HCC by regulating tumor microenvironment.

In this research, comprehensive bioinformatics methods and functional analysis were performed to reveal the SLC1A4 expression, prognostic role, and biological function of SLC1A4 in HCC, providing a novel prognostic biomarker and therapeutic strategy of HCC.

## Materials and Methods

### mRNA Expression

The gene RNA-sequencing dataset of HCC with related clinical data were downloaded from TCGA (https://cancergenome.nih.gov/) (10), ICGC (https://icgc.org/daco), GCPIA (https://icgc.org/daco) and GEO databases (https://www.ncbi.nlm.nih.gov/gds). The SLC1A4 expression in pan cancers was identified in the GEPIA database (http://gepia.cancer-pku.cn/) (11). GTEx (http://xena.ucsc.edu/) database was used to analyze SLC1A4 mRNA expression in normal tissues.

### Survival Analysis

The survival analysis of HCC patients in TCGA was performed via GEPIA database and Kaplan-Meier plotter (http://kmplot.com/analysis/) (12) and the survival analysis of HCC patients in ICGC was performed using R software. The multivariate or univariate cox regression models were performed using R software.

### Clinicopathological Characteristics

TCGA and ICGC databases were used to explore the correlation of SLC1A4 expression with clinicopathological factors of HCC using R software.

### Co-expressed Genes of SLC1A4

To explore SLC1A4 co-expressed genes in HCC, we performed volcano plots, heat maps, Pearson's correlation coefficient, or scatter plots using the LinkedOmics database. Gene Ontology (GO) and KEGG pathway analysis were performed using SLC1A4 related co-expressed genes.

### GSEA Enrichment Analysis

Basing on SLC1A4 expression, potential SLC1A4-regulated pathways were analyzed by GSEA. And pathways enriched in each phenotype were classified by Normalized enrichment score (NES) and *P*-value.

### Immune Infiltrates Analysis

Tumor Immune Estimation Resource (TIMER) database was used to reveal the immune invasion and gene expression in cancers (13). This study evaluated the expression of SLC1A4 in HCC and its association with immune cell infiltration via gene modules. Furthermore, CIBERSORT (14) was used to assess the association of immune response of immune cells with SLC1A4 expression in HCC. We analyzed SLC1A4 related immune markers using R software with *P* <0.05.

### Protein Expression

The Clinical Proteomic Tumor Analysis Consortium (CPTAC) proteomics database (https://cptac-data-portal.georgetown.edu/cptacPublic/) and IHC from the Human Protein Atlas (HPA) database (https://www.proteinatlas.org/) were used to assess SLC1A4 expression in HCC.

### Cancer Pathway Activity and Drug Sensitivity

GSCALite (http://bioinfo.life.hust.edu.cn/web/GSCALite/) (15) was used for SLC1A4-related cancer pathway activity and drug sensitivity via TCGA HCC dataset as previous described (16).

### qRT-PCR and Western Blot

SLC1A4 mRNA and protein expression in the HCC cells and normal liver cells were detected by Western blots and qRT-PCR, as described previously (17). The primary antibodies were anti-SLC1A4 (proteintech, 13067-2-AP, 1:100) and anti-GAPDH (proteintech, 60004-1-Ig, 1:5,000). The SLC1A4 primer used for the amplification was as follows: 5′- TGTTTGCTCTGGTGTTAGGAGT-3′ and 5′- CGCCTCGTTGAGGGAATTGAA-3′ (Sangon Biotech, China).

### Cell Culture and Small Interfering RNA Transfection

Human HCC cell lines Huh7 and LM3 were maintained with 5% CO_2_ at 37°C in DMEM (HyClone Germany) with 10% fetal bovine serum (GIBCO USA). Cells were transfected with siRNA (purchased from RIBOBIO) using Lipofectamine 2000. The targets of the SLC1A4 siRNAs were 5′- GTGTTAGGAGTGGCCTTAA−3′ and 5′- ACCCTTCCCTCTATGATGA−3′ (TSINGKE, China).

### Cell Migration Assay

Cell migration assays were performed as described previously (18).

### Cell Proliferation Assay

Cell proliferation ability was identified by CCK-8 assay as described previously. The assay was performed three times in triplicate (19).

### Cell-Cycle and Cell Apoptosis Assay

Cell apoptosis and cell-cycle were detected by Flow-cytometry analysis, as described previously (20).

### Clinical Samples and Immunohistochemistry

Fifteen formalin-fixed, paraffin-embedded HCC and paired adjacent liver tissues were collected from Xiangya Hospital of Central South University from 2017 to 2019. Our study was accepted by the ethics committee of Xiangya Hospital, Central South University.

According to previously described (21), IHC and the immunoreactive score of SLC1A4 (proteintech, 13067-2-AP, 1:100) were conducted on the formalin-fixed and paraffin-embedded tissue sections.

### Statistical Analysis

Statistical obtained from TCGA were all analyzed by R-3.6.1. The differential expression of the SLC1A4 in the TCGA, ICGC and GEO cohort were evaluated using the R package. The survival package was used for the survival analysis of the sample from ICGC. The relationship of SLC1A4 expression and clinical characteristics was assessed applying logistic regression. Univariate and multivariate analysis revealed the association of SLC1A4 with clinical factors, the immune infiltration with OS of HCC via survival R package. The ROC curves were analyzed using the survival ROC package. SPSS (ver 20.0, Inc., Chicago, IL, USA) and GraphPad Prism (7.0, La Jolla, CA, USA) were used to calculate other data. *P* < 0.05 was considered statistically significant.

## Result

### The Expression of SLC1A4 in HCC

**Figure 1:**
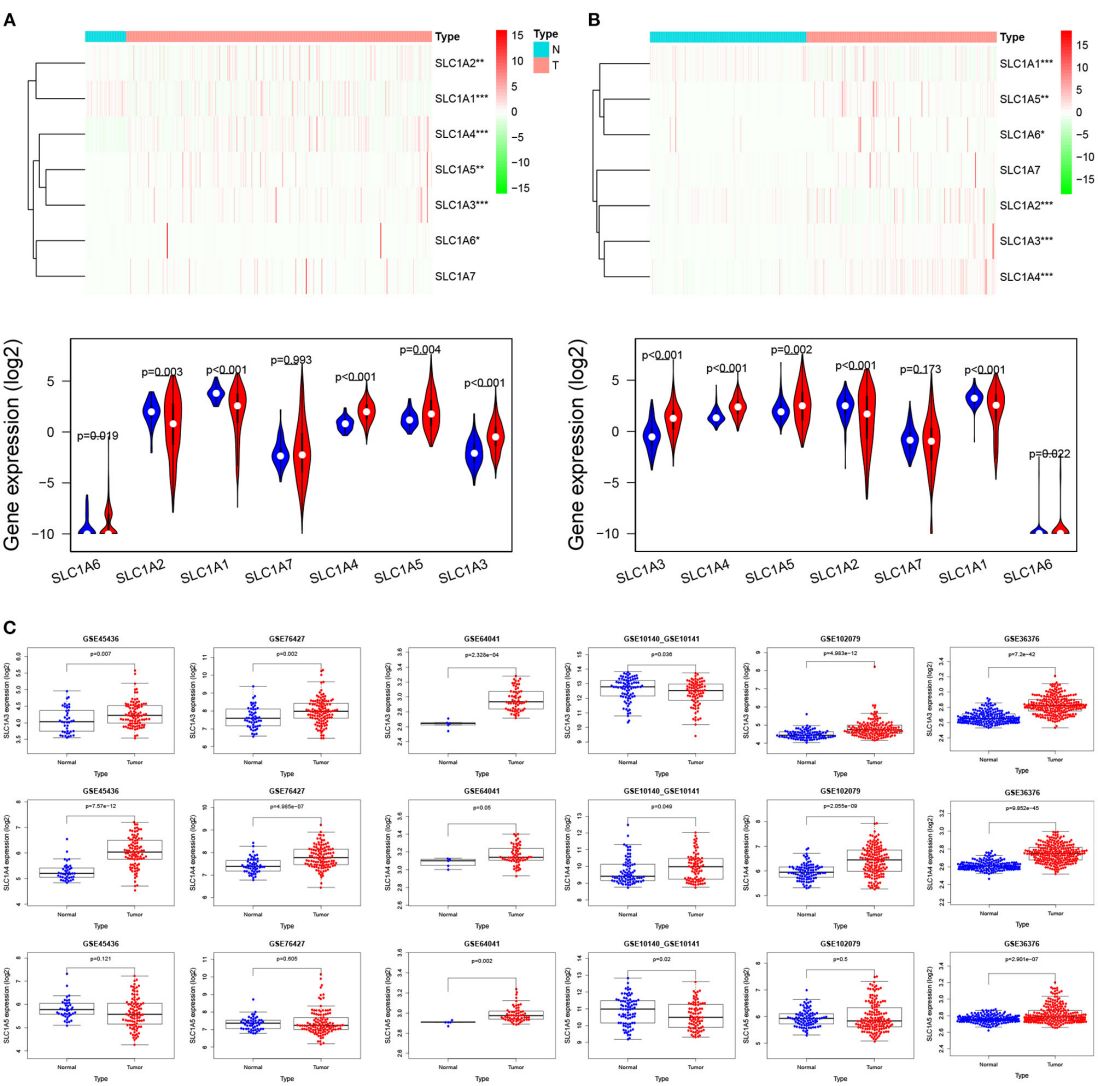
mRNA expression and prognosis of SLC1A4 in HCC. Comparison of SLC1A (1-7) mRNA expression in HCC tissues and normal tissues from TCGA **(A)**, ICGC **(B)** and GEO database **(C)**. *p < 0.05, **p < 0.01, ***p < 0.001.

**Figure 2:**
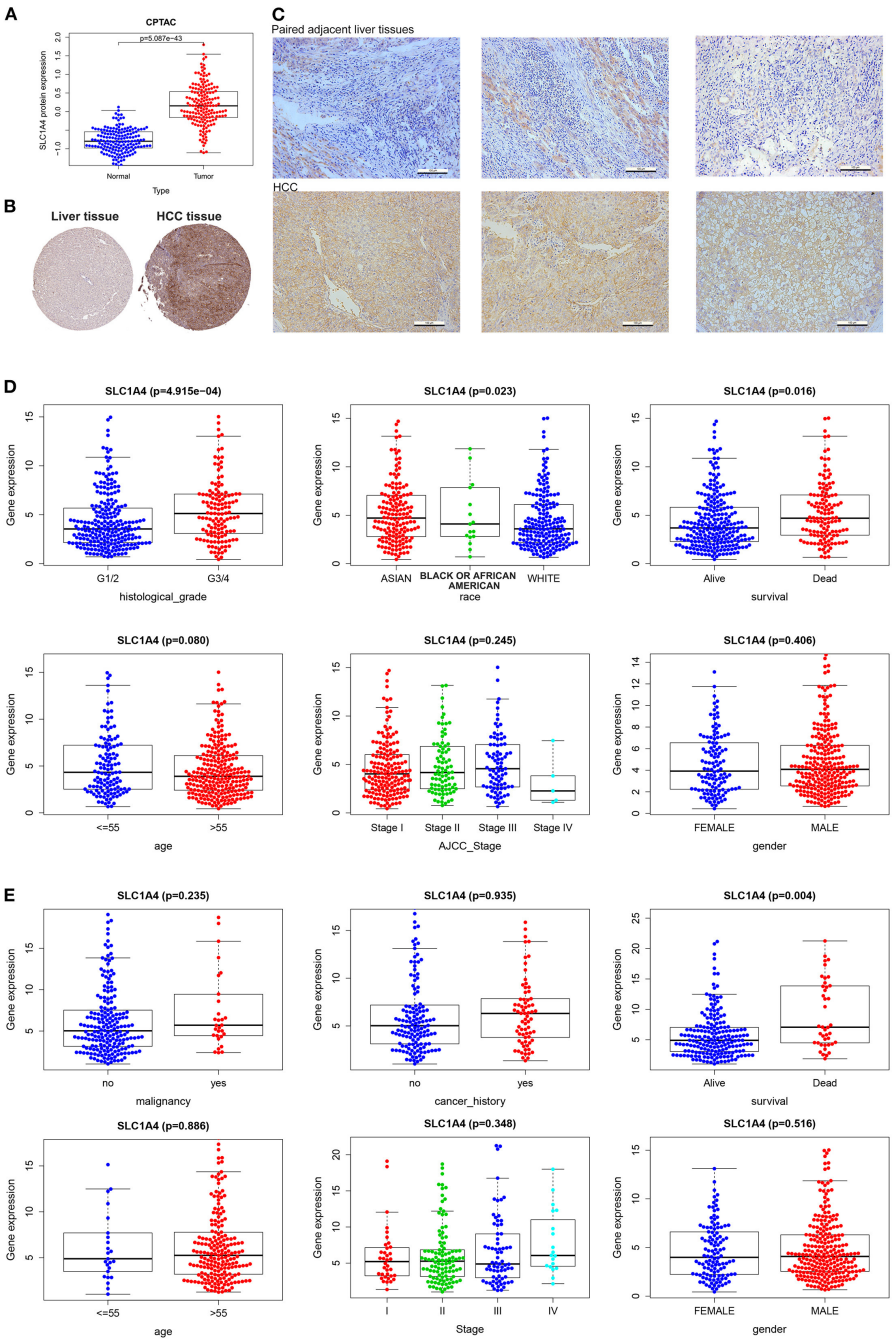
SLC1A4 expression and its correlation with the clinicopathological characteristics of HCC. SLC1A4 expression between abnormal and normal tissues in CPTAC **(A)** and HPA **(B)** databases. **(C)** The protein levels of SLC1A4 in 15 pairs of HCC tissue and compared normal tissue using IHC. The TCGA **(D)** and ICGC **(E)** databases revealed SLC1A4 mRNA in the various clinicopathologic feature.

We used multiple databases to analyze the mRNA expression of SLC1A family (SCL1A1-7) in HCC. As showed in Figures 1A,B, we observed SLC1A3, SLC1A4 and SLC1A5 mRNA expression was over-expressed in HCC tissues compared to normal tissues in TCGA and ICGC (LogFC>1, *p* < 0.01). We next analyzed SLC1A3-5 expression in HCC using 6 GEO datasets. As showed in Figure 1C, only SLC1A4 mRNA expression were steadily upregulated in HCC (GSE10140_GSE10141, GSE45436, GSE64041, GSE36376, GSE76427 and GSE102079 databases). So, SLC1A4 was selected for further analysis. The pancancer analysis showed that SLC1A4 mRNA was remarkably elevated in most tumor tissues, including BRCA, COAD, DLBC, ESCA, and so on (Supplementary Figure 1). Then, we utilized the GTEx database to explore SLC1A4 mRNA expression in normal tissues of male and female. Green represented low expression and red represented high expression. We observed that SLC1A4 was lowly expressed in most tissues, including the liver, whereas high expression of SLC1A4 was observed in adrenal_gland, brain, pancreas, and pituitary_gland of different genders (Supplementary Figures 2A,B). Interestingly, there were differentially expressed in males compared to females, such as bone_marrow, fallopian_tube, ovary, uterine_cervix, uterus, vagina, prostate, and testis. No significant difference of SLC1A4 expression present in liver tissues of males and females indicated that we could integrate liver tissues of different genders. CPTAC database analysis indicated that the protein level of SLC1A4 was up-regulated in HCC tissues compared to normal tissues (*p* = 5.087e-43) (Figure 2A). Using the HPA database, we also found that SLC1A4 protein expression was up-regulated in HCC tissue compared to normal liver tissue (Figure 2B). Moreover, we detected SLC1A4 expression in 15 pairs of HCC tissue and compared normal tissue using IHC. As shown in Figure 2C, SLC1A4 expression was significantly increased in HCC.

### Correlation of SLC1A4 Expression With Clinicopathological Characteristics

We investigated the correlation between SLC1A4 expression with the clinicopathological feature in HCC. Over-expressed SLC1A4 was significantly correlation with histological_grade (*p* = 4.915e-04), race (*p* = 0.023), survival (*p* = 0.016) using TCGA database (Figure 2D). And there was a significant association between SLC1A4 with survival (*p* = 0.004) using the ICGC database (Figure 2E). As showed in Table 1, Kaplan-Meier plotter analysis indicated that sex, stage, virus hepatitis, alcohol consumption, sorafenib treatment, Race and vascular invasion adversely affected Overall survival and Progression-free survival. Specifically, male, stage (2, 1+2, 3, 2+3, and 3+4), grade (1 and 2), AJCC_T (2 and 3), Vascular invasion (micro), Race (White and Asian), Sorafenib treatment, Alcohol consumption, and no virus hepatitis adversely affected Overall survival but not Progression-free survival. Our results implied that over-expressed SLC1A4 is associated with shorter survival time of HCC patients.

**Table 1:** Kaplan-Meier plotter analysis of clinicopathological factors for overall survival and Progression-free survivalin HCC patients.

**Clinicopathological factors**	**Overall survival**			**Progression-free survival**		
	***N***	**Hazard ratio**	***P*-value**	***N***	**Hazard ratio**	***P*-value**
**Sex**						
Female	118	2.53(1.37–4.67)	**0.0023**	120	2.11(1.24–3.59)	**0.0047**
Male	246	1.82(1.16–2.84)	**0.0081**	246	0.8(0.55–1.16)	0.2297
**Stage**						
1	170	1.79(0.97–3.31)	0.0589	170	1.58(0.93–2.7)	0.0872
2	83	7.99(1.08–59.09)	**0.0155**	84	0.57(0.31–1.03)	0.0577
1+2	253	1.75(1.02–3.01)	**0.0392**	254	1.21(0.79–1.85)	0.3853
3	83	3.15(1.46–6.77)	**0.002**	83	1.62(0.94–2.78)	0.0768
2+3	166	2.88(1.6–5.2)	**0.0002**	167	1.22(0.82–1.82)	0.3209
4	4			5		
3+4	87	2.47(1.32-4.61)	**0.0035**	88	1.67(0.97–2.86)	0.0599
**Grade**						
1	55	3.25(1.25–8.47)	**0.0109**	55	1.56(0.65–3.74)	0.3133
2	174	2.52(1.38–4.62)	**0.0019**	175	1.56(0.97–2.5)	0.0618
3	118	1.82(0.93–3.56)	0.0751	119	0.69(0.38–1.23)	0.2081
4	12			12		
**AJCC_T**						
1	180	1.7(0.95–3.07)	0.0728	180	1.55(0.94–2.58)	0.0854
2	90	4.76(1.13–20.07)	**0.0188**	92	0.66(0.38–1.14)	0.13
3	78	2.55(1.18–5.51)	**0.0136**	78	1.48(0.83–2.65)	0.1858
4	13			13		
**Vascular invasion**						
micro	90	2.32(1.09–4.95)	**0.0255**	91	0.67(0.38–1.19)	0.168
macro	16			16		
None	204	1.5(0.87–2.58)	0.1375	204	1.32(0.83–2.08)	0.2384
**Race**						
White	181	1.66(1.03–2.66)	**0.0355**	183	1.48(0.97–2.26)	0.0685
Asian	155	2.74(1.41–5.33)	**0.0019**	155	1.82(0.97–3.39)	0.0566
Black or African American	17			17		
**Sorafenib treatment**						
Treated	29	4.18(0.9–19.36)	**0.0484**	30	1.99(0.88–4.48)	0.092
**Alcohol consumption**						
yes	115	2.54(1.11–5.79)	**0.0221**	115	1.26(0.71–2.23)	0.4359
none	202	1.9(1.12–3.23)	**0.0162**	204	1.25(0.82–1.91)	0.3003
Virus hepatitis						
Yes	150	2.13(1.08–4.18)	**0.0255**	152	0.67(0.4–1.12)	0.121
None	167	2.06(1.3–3.26)	**0.0017**	167	1.68(1.06–2.65)	**0.0251**

*The meaning of the bold values is P-value < 0.05*.

### The Correlation Between SLC1A4 Expression and Prognosis in HCC Patients

**Figure 3:**
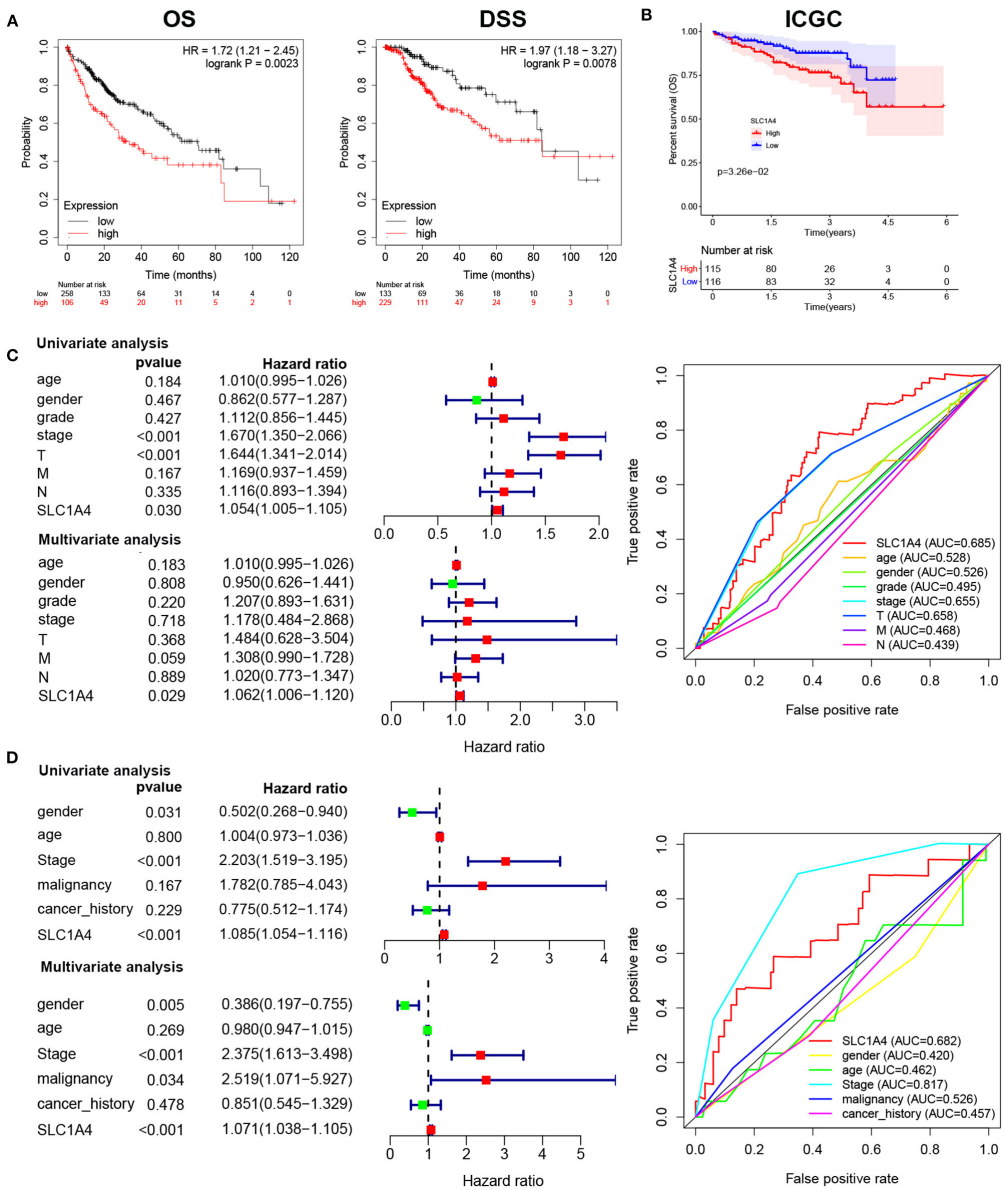
Expression of SLC1A4 was correlated with prognosis of HCC patients. The association of SLC1A4 with **(A)** overall survival and disease-specific survival in HCC using GEPIA database, **(B)** overall survival from ICGC database. Univariate and multivariate Cox regression analysis and ROC curve identified the association of SLC1A4 with the clinical factors with OS in **(C)** TCGA database and **(D)** ICGC database.

We next analyzed the prognostic role of SLC1A4 in HCC. GEPIA analysis showed HCC patients with high expressed SLC1A4 shows poor OS and DSS (Figure 3A). In addition, high expressed SLC1A4 correlated with worse OS of HCC in the ICGC database (Figure 3B). Then, univariate and multivariate Cox regression analysis indicated SLC1A4 was a significant prognostic marker for OS in the TCGA database (Figure 3C). Furthermore, stage, SLC1A4, and malignancy were a significant prognostic marker for OS in the ICGC database (Figure 3D). The results suggested that SLC1A4 expression in HCC was regarded as a potential prognostic marker of OS.

### Potential Pathway Regulated by SLC1A4 in HCC

**Figure 4:**
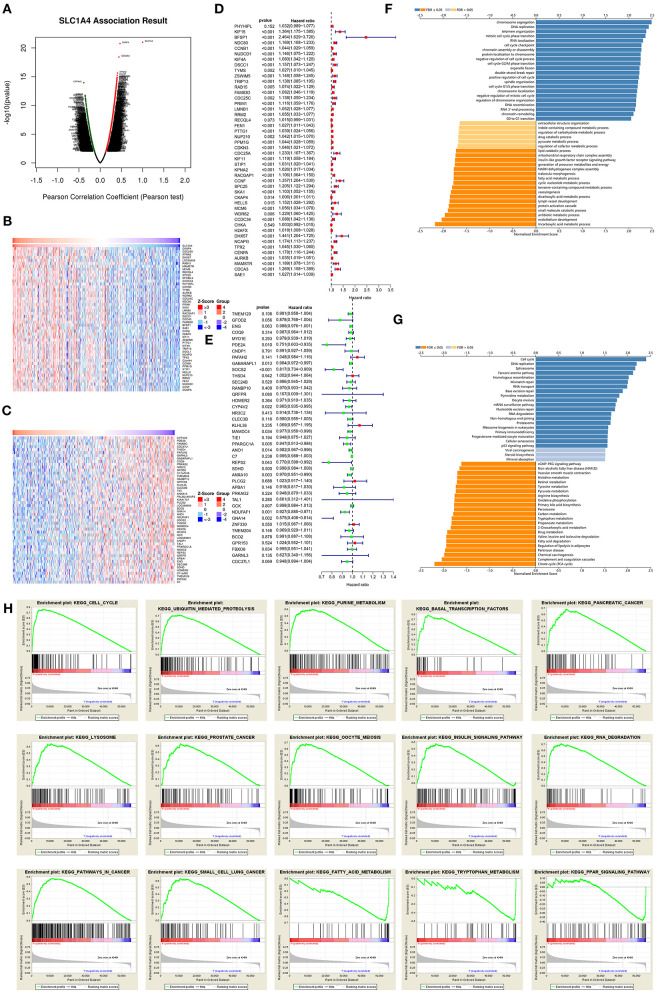
The potential SLC1A4-regulated pathway. **(A)** LinkedOmics was used to analyzed co-expressed genes of SLC1A4 in HCC. A Pearson test was used to analyze the association of SLC1A4 with genes expressed in HCC cohort. Heat maps showing genes positively **(B)** and negatively **(C)** related to SLC1A4 in the HCC (TOP 50). The high-risk genes in positive genes **(D)** and low-risk genes in negatively significant genes **(E)** (TOP 50). GO annotations **(F)** and KEGG pathways **(G)** of SLC1A4 in the HCC cohort. **(H)** GSEA analysis revealed the pathway enriched in SLC1A4 high and low expression phenotype.

Gene co-expression reflected common genetic risk factors constituting functional relationships. Thus, we used LinkedOmics to detect co-expressed genes of SLC1A4 in HCC. As showed in Figure 4A, (2946) genes (dark red dots) revealed a significant positive association with SLC1A4, while (2179) genes (dark green dots) indicated significantly negative associations. The heat map showed that the top 50 significant genes were positively and negatively associated with SLC1A4 (Figures 4B,C). Especially, there were 43 of the top 50 prominently positive genes were high-risk genes (Figure 4D), and 11 of the top 50 negatively significant genes were low-risk genes (Figure 4E). GO analysis and KEGG pathway analysis showed that SLC1A4 may be involved in the regulation of cell cycle, DNA replication, chromosome segregation and RNA localization, etc (Figures 4F,G). These results implied that SLC1A4 might widely impact on the transcriptome in HCC. The sample was divided into a low-expressed and high-expressed SLC1A4 group. And the GSEA analysis indicated that ubiquitin-mediated proteolysis, cell cycle, purine metabolism, basal transcription factors, pancreatic cancer, lysosome, prostate cancer, oocyte meiosis, pathways in cancer, small cell lung cancer, RNA degradation and insulin signaling pathway were enrichment in SLC1A4 high-expressed group (Figure 4H). On the contrary, tryptophan metabolism, fatty acid metabolism, and PPAR signaling pathway were enrichment in the SLC1A4 low-expressed group (Figure 4H). However, the potential pathway regulated by SLC1A4 need to be further approved by experimental data.

### The Correlation of SLC1A4 Expression and Tumor Immune Infiltrations

**Figure 5:**
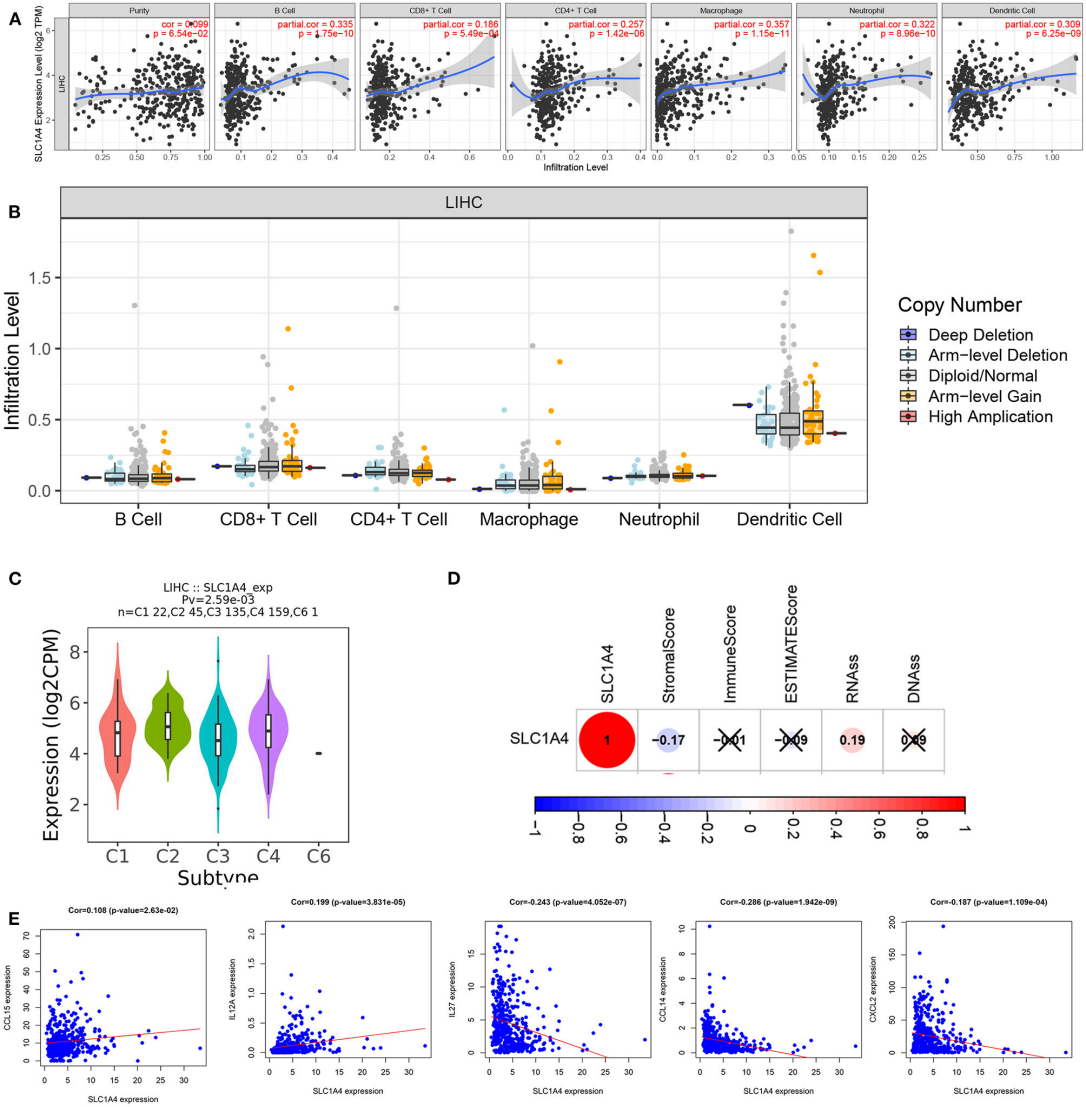
The association of SLC1A4 with immune infiltration in HCC. **(A)** SLC1A4 expression is significantly associated with tumor purity and infiltrating of immune cells. **(B)** SLC1A4 CNV affects the infiltrating of immune cells. **(C)** The association between SLC1A4 expression and immune subtype using TISIDB, and **(D)** immune score and Tumor stem cell score. **(E)** The association between SLC1A4 and HCC-related chemokines.

Next, we explored whether SLC1A4 was associated with immune infiltration in HCC. As showed in Figure 5A, SLC1A4 expression was significant associations with tumor purity (*p* = 6.54e-02), B cell (*p* = 1.75e-10), neutrophils (*p* = 8.96–10), macrophages (*p* = 1.15e-11), CD4+ T cells (*p* = 1.42e-06), CD8+ T cells (*p* = 5.49e-04) and dendritic cells (*p* = 6.25e-09) in HCC. However, there were no significant correlations of SLC1A4 CNV with all immune cells in HCC (Figure 5B). Furthermore, SLC1A4 expression was significant associated with immune subtypes using TISIDB (Figure 5C). We next analyzed the association between SLC1A4 and tumor microenvironment, tumor stem cell score in HCC. And SLC1A4 was significantly associated with the stroma score and tumor stem cell RNAs score (Figure 5D). However, the potential correlation of SLC1A4 expression and tumor immune infiltrations need to be further approved by experimental data.

To further investigate the correlations between SLC1A4 expression with the diverse immune infiltrating cells, we analyzed the association of SLC1A4 with immune markers via the GEPIA database. Multiple immune cells were detected, including T cell (general), B cell, CD8+ T cell, T cell exhaustion, Th17, Tfh, Th1, Neutrophils, Natural killer cell, Th2, and Mast cells (Table 2). The results showed that CD8A, CD8B, CD2, CD3E, CD3E, KIR2DL1, KIR2DL3, KIR2DL4, KIR3DL2, KIR2DS4, CD11b, T-bet, STAT6, STAT5A, BCL6, STAT3, CTLA4, LAG3 and TIM-3 had a significant association with SLC1A4 expression level in tumor tissue from HCC patients. Meanwhile, neutrophils markers (CD11b), Th1 markers (T-bet), Th2 markers (STAT6 and STAT5A) and Th17 markers (STAT3) indicated a significant association with the expression of SLC1A4 in normal tissue from HCC patients. And natural killer cell markers including KIR3DL1, Neutrophils markers (CD11b and CCR7), Th2 markers (STAT6, STAT5A and IL13), Tfh markers (BCL6), Th17 markers (STAT3), T cell exhaustion markers (LAG3 and TIM-3), Mast cells markers (TPSB2, TPSAB1, CPA3 and MS4A2) were also significantly correlated with the SLC1A4 expression in normal liver tissue from GTEx. According to these results, SLC1A4 played a crucial role in immune activities in the tumor microenvironment. We also evaluated the association between SLC1A4 and immune markers in HCC tissue from ICGC datasets (Supplementary Figure 3). The heat map showed that SLC1A4 moderately associated with the expression of CD8A, CD8B, CD2, CD3E, KIR2DL4, ITGAM, STAT5A, BCL6, CTLA4 and LAG3. Moreover, SLC1A4 was also associated with the HCC-related chemokines including CCL15, CCL14, CXCL2, IL12A and IL27 (Figure 5E).

**Table 2:** The association of SLC1A4 expression and immune markers in GEPIA.

**Description**	**Gene markers**	**HCC**					
		**Tumor**		**Normal**		**GTEX**	
		**R**	**P**	**R**	**P**	**R**	**P**
CD8+ T cell	CD8A	0.16	0.0028	0.11	0.44	−0.0022	0.98
	CD8B	0.16	0.0023	0.14	0.34	−0.004	0.97
T cell (general)	CD2	0.13	0.013	0.083	0.57	0.014	0.88
	CD3E	0.12	0.017	0.12	0.42	−0.018	0.85
B cell	CD19	0.061	0.24	0.21	0.13	0.0029	0.98
	CD79A	0.069	0.19	0.19	0.18	0.087	0.36
Natural killer cell	KIR2DL1	0.13	0.015	0.019	0.9	−0.075	0.44
	KIR2DL3	0.14	0.0063	−0.028	0.85	−0.14	0.16
	KIR2DL4	0.12	0.017	−0.0024	0.99	0.015	0.87
	KIR3DL1	0.098	0.06	0.011	0.94	−0.19	0.048
	KIR3DL2	0.13	0.016	0.037	0.8	0.014	0.89
	KIR3DL3	0.098	0.06	−0.026	0.86	0.15	0.11
	KIR2DS4	0.16	0.0016	−0.042	0.77	−0.11	0.24
Neutrophils	CD66b	−0.0052	0.92	0.12	0.4	−0.034	0.73
	CD11b	0.2	1.50E-04	0.31	0.028	0.29	2.10E-03
	CCR7	0.055	0.29	0.16	0.28	0.42	4.80E-06
Th1	T-bet	0.13	0.013	0.31	2.80E-02	−0.16	0.1
	STAT4	0.077	0.14	0.19	0.18	0.082	0.39
	TNF-α(TNF)	0.062	0.23	0.28	0.051	0.041	0.67
Th2	GATA3	0.098	0.06	0.2	0.16	0.077	0.42
	STAT6	0.16	0.0015	0.49	3.40E-04	0.16	9.50E-02
	STAT5A	0.23	7.30E-06	0.37	8.00E-03	0.47	2.00E-08
	IL13	−0.036	0.49	−0.015	0.92	−0.2	0.032
Tfh	BCL6	0.27	1.20E-07	0.22	0.13	0.51	1.40E-08
Th17	STAT3	0.21	3.20E-05	0.33	0.018	0.59	1.10E-11
	IL17A	0.093	0.074	0.054	0.71	−0.011	0.91
T cell exhaustion	PD-1(PDCD1)	0.078	0.13	0.077	0.59	0.017	0.86
	CTLA4	0.16	0.0027	−0.028	0.85	0.02	0.84
	LAG3	0.17	1.10E-03	−0.025	0.86	−0.39	2.50E-06
	TIM-3	0.076	1.50E-01	0.25	0.085	0.27	4.20E-03
Mast cells	TPSB2	−0.031	0.55	0.16	0.27	−0.28	0.0035
	TPSAB1	−0.044	0.4	0.22	0.12	0.24	0.012
	CPA3	−0.0063	0.9	0.15	0.28	−0.21	0.028
	MS4A2	−0.0089	0.86	0.23	0.1	−0.27	0.0048
	HDC	−0.077	0.14	−0.013	0.93	−0.0074	0.94

### Silenced SLC1A4 Repressed HCC Progression and Immune-Related Chemokines Expression

**Figure 6:**
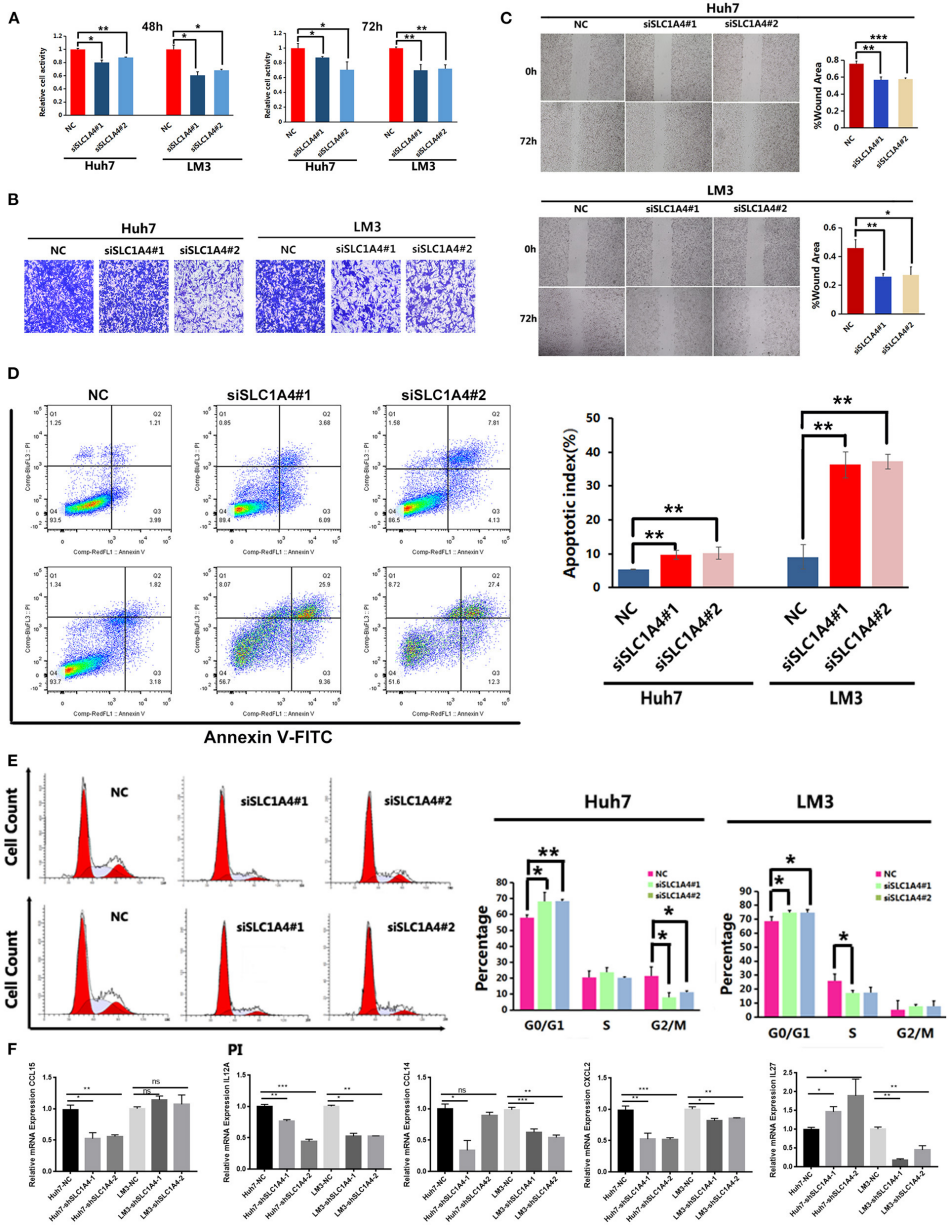
The effects of SLC1A4 on HCC cell proliferation, migration, apoptosis and cell cycle. **(A)** MTT analysis revealed the effects of silenced SLC1A4 on cell proliferation **(B)** Transwell analysis and **(C)** scratch wound healing assay revealed the migration of HCC cells. The flow cytometric analysis was carried out to analysis the cell apoptosis **(D)** and Cell cycle distribution **(E)** in HCC cells. **(F)** Immune-related chemokines expression in SLC1A4 silenced HCC cells. *p < 0.05, **p < 0.01, ***p < 0.001.

Next, two SLC1A4 siRNAs were used to reveal the role of SLC1A4 on the progression of HCC. As shown in Supplementary Figure 4, SLC1A4 siRNA evidently reduced SLC1A4 expression in both Huh7 and LM3 cells. CCK8 analysis showed that silenced SLC1A4 evidently reduced cell viability in HCC cells (Figure 6A). Transwell migration analysis and wound healing analysis showed that silenced SLC1A4 evidently reduced cell migration ability in two cells (Figures 6B,C). Next, the flow cytometric analysis was carried out to reveal the effects of SLC1A4 on cell apoptosis and cell cycle. As shown in Figure 6D, the number of total apoptotic SLC1A4 silenced Huh7 and LM3 cells were significantly up-regulated compared to that of the NC group. And the number of SLC1A4 silenced Huh7 and LM3 cells in the G1-stage was higher than that in the NC group (Figure 6E). In short, these results showed that SLC1A4 participated in the progression of HCC via regulating cell migration, proliferation, apoptosis and cell cycle of Huh7 and LM3 cells.

Moreover, we analyzed the immune-related chemokines expression in SLC1A4 silenced HCC cells. As shown in Figure 6F, CCL15 was downregulated in SLC1A4 silenced Huh7 cell; CCL14, CXCL2 and IL12A were downregulated in both SLC1A4 silenced Huh7 and LM3 cells; IL27 were downregulated in SLC1A4 silenced Huh7 cell and upregulated in SLC1A4 silenced LM3 cell. These results indicated that SLC1A4 could affect immune cell infiltration partly by regulating these chemokines expression.

### Drug Sensitivity and Cancer Pathway Activity

**Figure 7:**
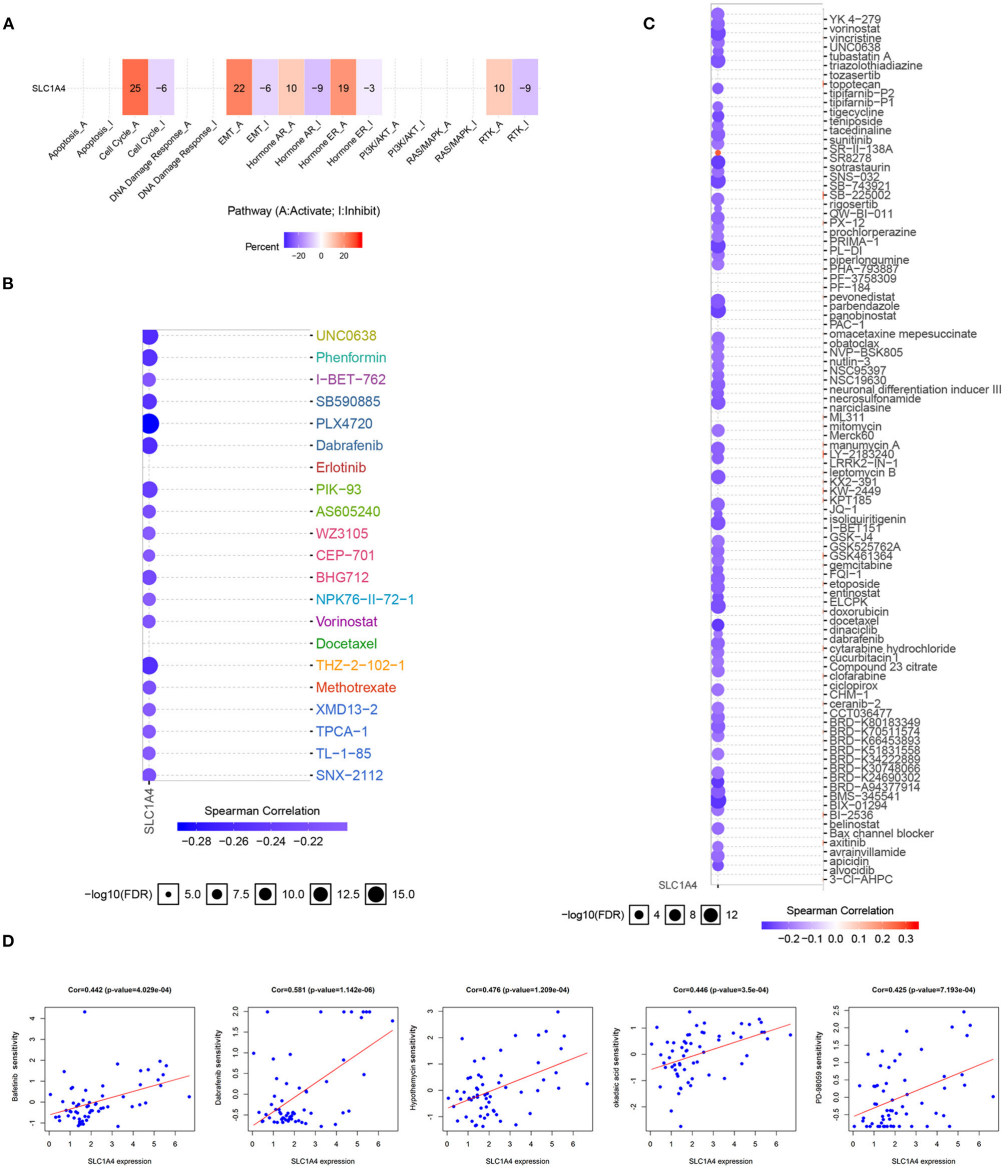
The correlation of drug sensitivity and Cancer pathway activity and SLC1A4 in HCC patients. **(A)** SLC1A4-related Cancer pathway activity. SLC1A4-related drug sensitivity using GDSC **(B)** and CTRP **(C)** database. **(D)** The association between SLC1A4 and drug sensitivity.

According to their crucial clinical value, the drug sensitivity of SLC1A4 in HCC patients was analyzed. As shown in Figure 7A, SLC1A4 was primarily participated in the activation of the cell cycle, RTK pathways, Hormone ER pathway, and EMT pathway. In addition, low-expressed SLC1A4 exhibited resistance to 21 and 69 drugs in GDSC and CTRP, respectively, using drug sensitivity analysis (Figures 7B,C). Moreover, the correction of SLC1A4 expression and drug sensitivity (Bafetinib, Dabrafenib, Hypothemycin, okadaic acid and PD-98095) were observed in Figure 7D. These results provide novel and optional therapy strategies for HCC patients with high expressed SLC1A4.

## Discussion

Hepatocellular carcinoma is a major health problem, leading to the second most common cause of tumor-related death. Although treatment strategies for HCC have greatly improved over recent decades, the survival rate is still undesirable. Thus, it is imperative to develop novel prognosis biomarkers and therapeutic targets for HCC (1, 2, 22). In our research, multiple bioinformatics analysis combined with functional analysis methods were applied to explore the expression, prognostic role, and biological function of SLC1A4 in HCC.

As a Na-dependent amino acid transporter, SLC1A4 has been characterized in both neuronal and non-neuronal cells and tissues. To explore the latent role of SLC1A4 and its regulatory network in HCC progression and immunity, we applied a multiple-dimensional bioinformatics analysis and carried out the subsequent functional verification, which can provide a direction for future in-depth research related to HCC. At first, we analyzed the expression of all the members in the SLC1A family (SCL1A1-7) using TCGA and ICGC databases. SLC1A4 was selected for further analysis (see Supplementary Data). Next, TCGA, ICGA, GCPIA, and GEO databases were used to investigate the prognostic value of SLC1A4 in HCC patients. Overexpressed SLC1A4 was observed in HCC patients and significantly correlated with worse overall and disease-specific survival of HCC patients. The HCC patients with overexpressed SLC1A4 are more likely to observe advanced stage, histological grade, poor prognosis, and macro-vascular invasion than low-expressed SLC1A4. Furthermore, the functional analysis revealed that silenced SLC1A4 inhibited HCC cell proliferation, migration, and induced HCC cell apoptosis. To explore the SLC1A4-mediated regulatory network in HCC, the co-expressed genes were mostly associated with various pathways including repair and DNA replication, cell cycle checkpoint and metabolism. GSEA analysis showed that overexpressed of SLC1A4 participated in multiple carcinogenesis-associated signaling pathways and processes, including ubiquitin-mediated proteolysis, cell cycle, pancreatic cancer, lysosome, prostate cancer, RNA degradation, pathways in cancer and small cell lung cancer. In brief, these results suggest that SLC1A4 is a novel prognostic biomarker and therapeutic target of HCC.

The liver is a critical immune tissue and its lymphocyte cells are related to the biological and pathological processes of liver diseases (23). HCC is typical cancer in a background of chronic inflammation and immune dysregulation (24, 25). The treatment and prognosis of HCC are also associated with immune cell infiltration and function (24). Here, we provided proof of the relationship between SLC1A4 and immune infiltration in HCC. The expression of SLC1A4 significantly correlated with immune cells infiltration in HCC including B cell, neutrophils, macrophages, CD4+ T cells, CD8+ T cells and dendritic cells. SLC1A4 expression was also significantly associated with immune subtype, stroma score and tumor stem cell RNAs score. It is well-accepted that chemokines play key role in recruiting and positioning of immune cells in the tumor microenvironment (26–29). For example, CCL15 and IL12A were reported to play an important role on recruited CCR1+ CD14+ monocytes and natural killer (NK) cell in HCC respectively (30, 31). In this study, we found the positive correlation between SLC1A4 with CCL15 and IL12A (Figure 5E). And silenced SLC1A4 evidently reduced CCL15 and IL12A expression in HCC cells. SLC1A4 showed negative correlation with chemokines expression including CCL14, CXCL2 and IL27. CXCL2 is one of the major regulators for neutrophils recruitment, which plays a key role on regulating HCC progression (28). CCL14 is a tumor suppressor of HCC by modulating cell cycle and promoting apoptosis (32). IL-27 is well-known for its antitumor immunity in HCC (33). We found that silenced SLC1A4 evidently reduced these chemokines expression. Hence, we speculated that SLC1A4 affected neutrophils and monocytes infiltration partly by regulating CCL15 and CXCL2 expression in HCC cells.

Finally, drug sensitivity analysis showed that low-expression of SLC1A4 exhibited resistance to 21 and 69 drugs, a positive correction was also observed between SLC1A4 expression and drug sensitivity (Bafetinib, Dabrafenib, Hypothemycin, okadaic acid and PD-98095), suggesting that the five sensitivity drugs may be effective therapeutic strategy for HCC patients with high-expressed SLC1A4.

In brief, our research provided multi-levels evidence to confirm that SLC1A4 is a potential prognostic and therapeutic biomarker for HCC progression and it could affect immune microenvironment, partly by regulating chemokine expression in HCC cells, and the sensitivity drugs could provide novel therapeutic strategy for HCC. However, the regulation of SLC1A4 on immune microenvironment need to be further confirmed by experimental data.

## Data Availability Statement

The datasets presented in this study can be found in online repositories. The names of the repository/repositories and accession number(s) can be found in the article/Supplementary Material.

## Ethics Statement

The studies involving human participants were reviewed and approved by The ethics committee of Xiangya Hospital, Central South University. The patients/participants provided their written informed consent to participate in this study. Written informed consent was obtained from the individual(s) for the publication of any potentially identifiable images or data included in this article.

## Author Contributions

YZ and XP: conceptualization. RC, SL, SC, and XP: methodology. SL and XP: investigation. YZ and SL: writing – original draft. YZ, RC, and SL: writing – review & editing. YZ and RC: funding acquisition. All authors contributed to the article and approved the submitted version.

## Conflict of Interest

The authors declare that the research was conducted in the absence of any commercial or financial relationships that could be construed as a potential conflict of interest.
