# Supplementary material for: SLC1A4: A Powerful Prognostic Marker and Promising Therapeutic Target for HCC
Source: Front Oncol. 2021 Mar 11;11:650355. doi: 10.3389/fonc.2021.650355 (PMC7991385; doi:10.3389/fonc.2021.650355)
Supplement: Supplementary file 1 [file Data_Sheet_1.docx]

Supplementary Material


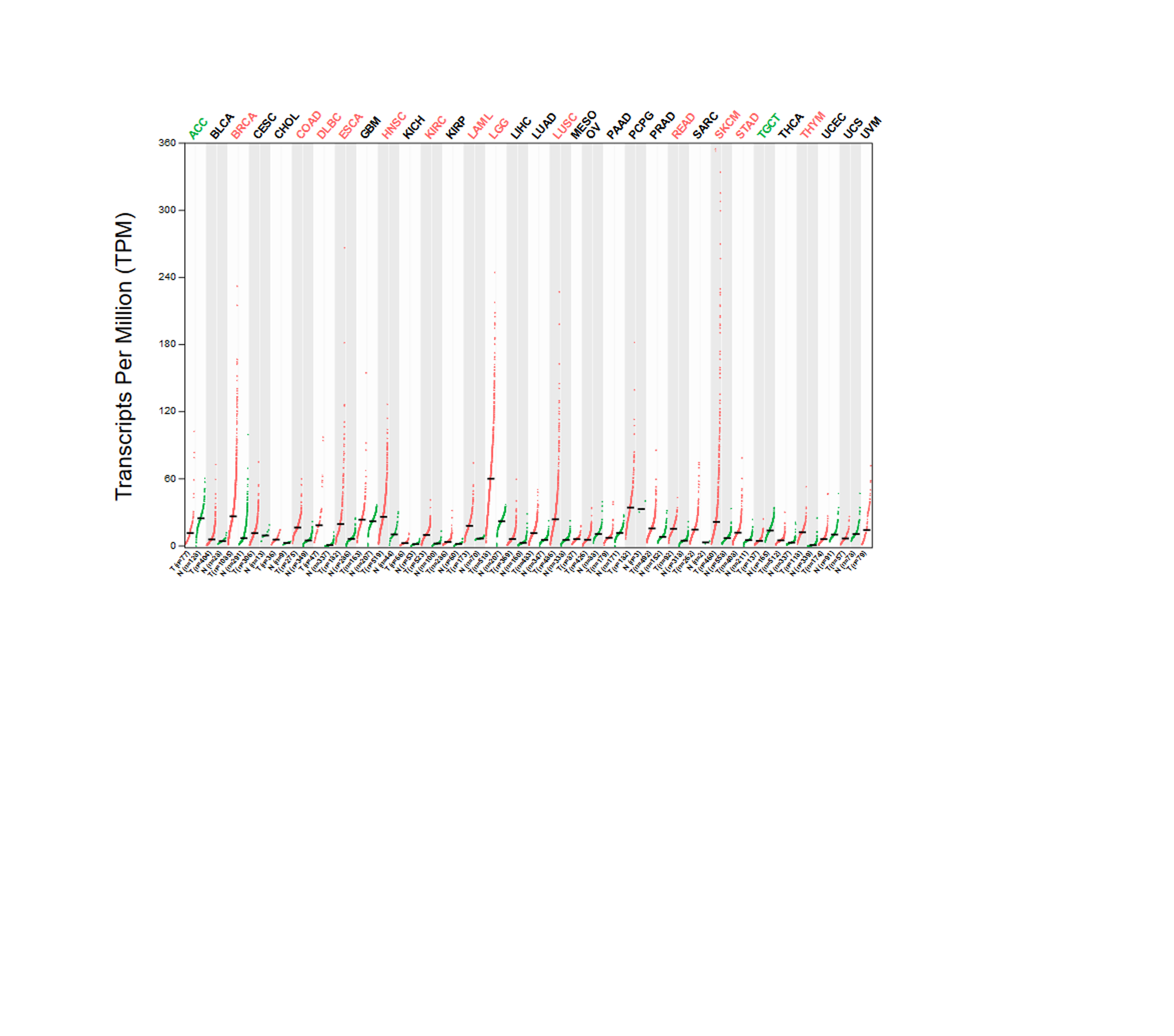


**Figure S1.** Expression of SLC1A4. SLC1A4 in multi-tumor tissues compared to adjacent normal tissues via GEPIA database.


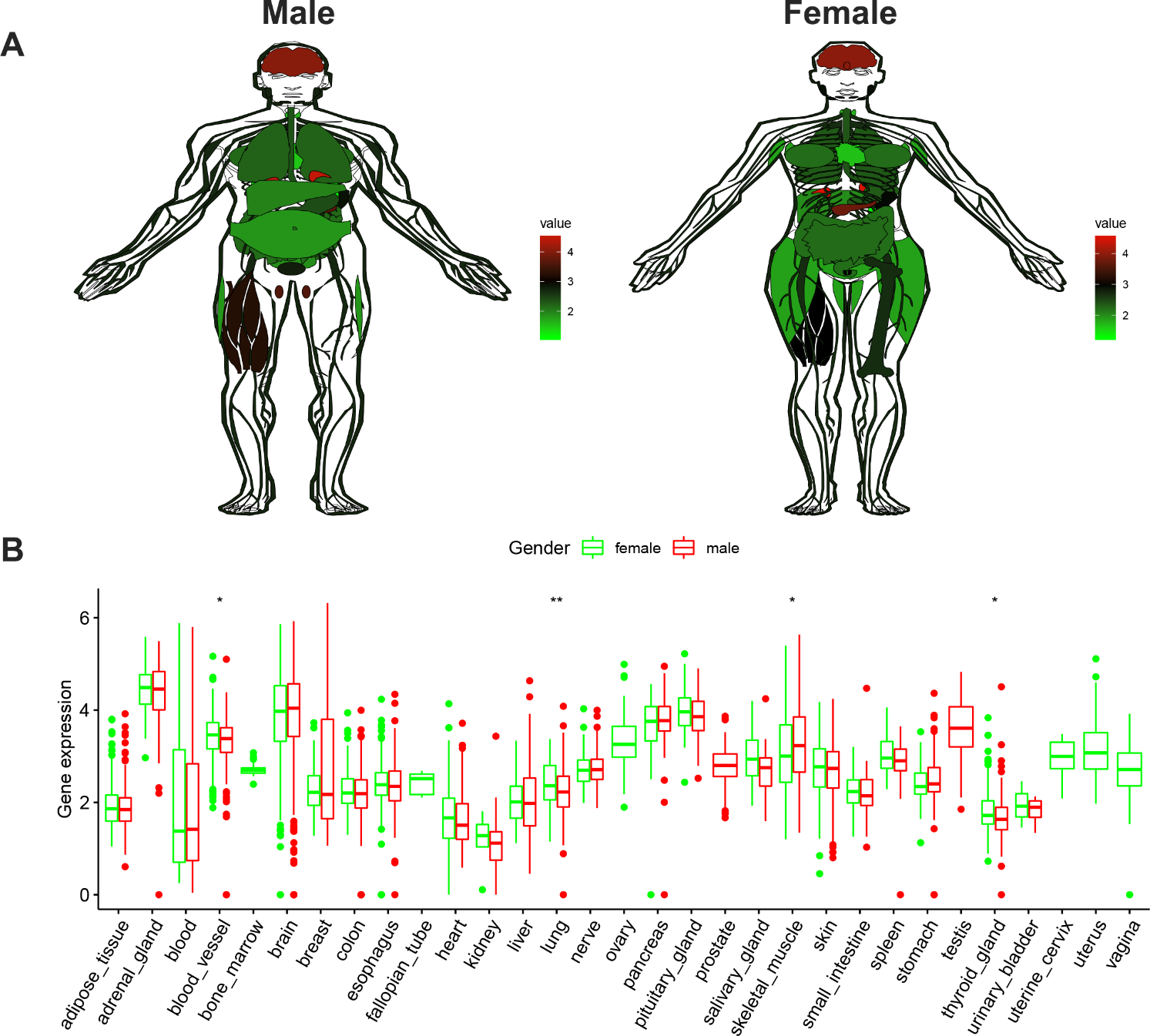


**Figure S2.** GTEx analyses of SLC1A4 expression in normal tissues (A). SLC1A4 expression in tissues of female and male (B).


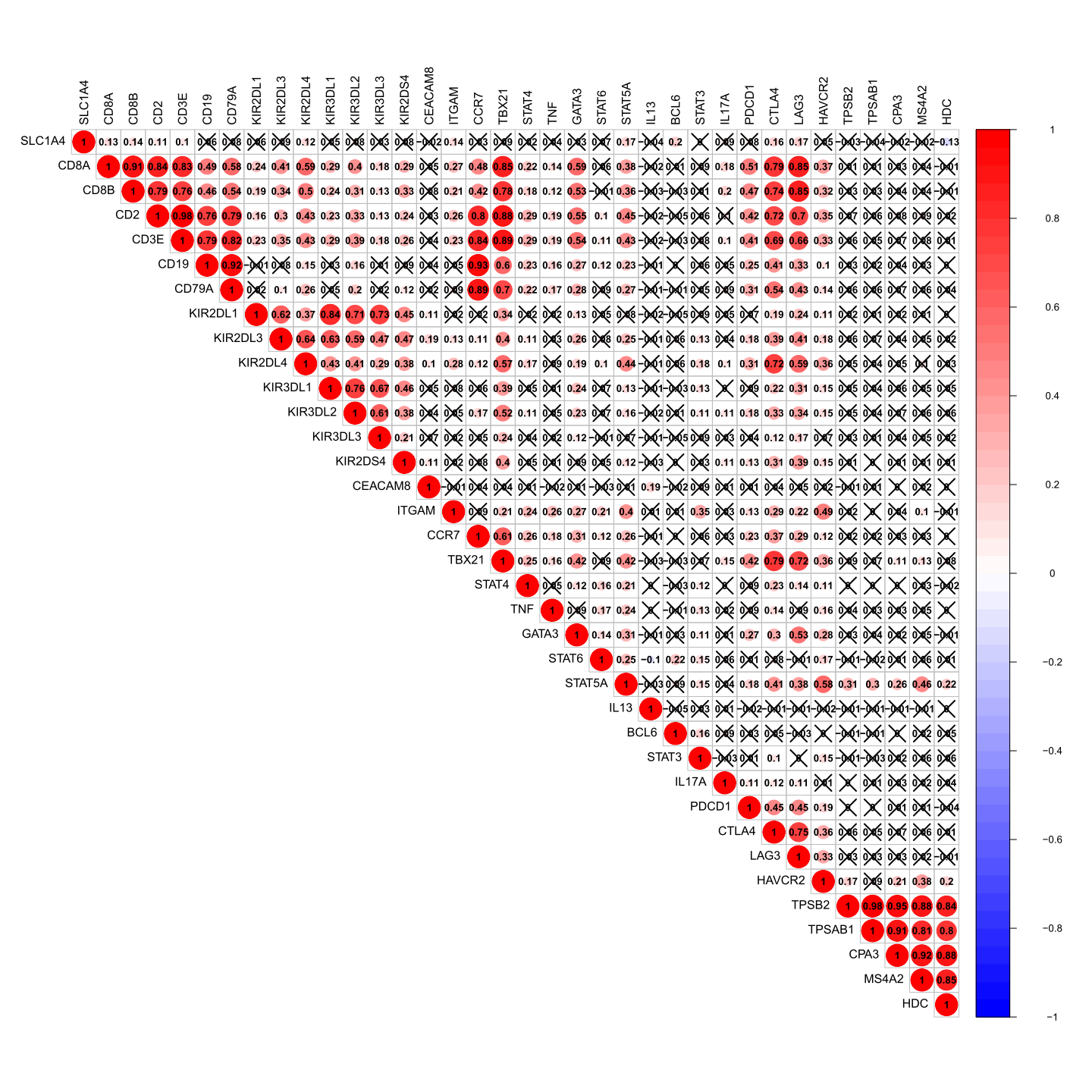


**Figure S3.** The correlation of SLC1A4 expression and immune markers.


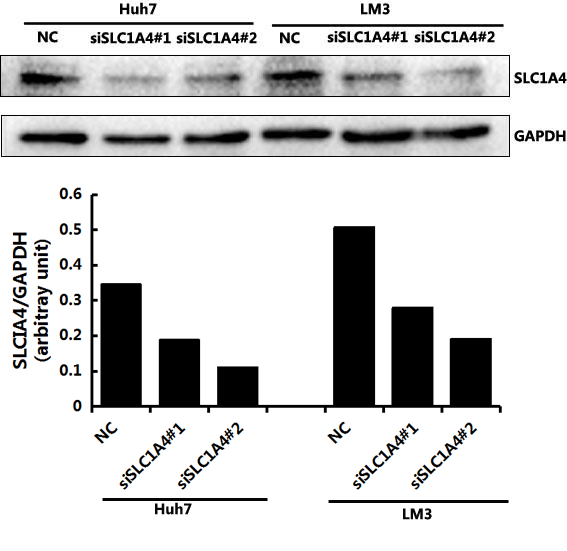


**Figure S4.** The protein expression of SLC1A4 in Huh7 and LM3 cells.

Table S1. The primers for qPCR

| **GENES** | **Primers (5' to 3')** |
| --- | --- |
| CCL15-F | TCCCAGGCCCAGTTCACAAAT |
| CCL15-R | TGCTTTGTGAGATGTAGGAGGT |
| IL12A-F | TGCCTTCACCACTCCCAAAACC |
| IL12A-R | CAATCTCTTCAGAAGTGCAAGGG |
| IL-27-F | GGAATCTCACCTGCCAGGAGTG |
| IL-27-R | TGGTGGAGATGAAGCAGAGACG |
| CCL14-F | CCAAGCCCGGAATTGTCTTCA |
| CCL14-R | GGGTTGGTACAGACGGAATGG |
| CXCL2-F | GGCAGAAAGCTTGTCTCAACCC |
| CXCL2-R | CTCCTTCAGGAACAGCCACCAA |
| GAPDH-F | GTCTCCTCTGACTTCAACAGCG |
| GAPDH-R | ACCACCCTGTTGCTGTAGCCAA |
